# Ultra-High Mass-Loading Cathode for Aqueous Zinc-Ion Battery Based on Graphene-Wrapped Aluminum Vanadate Nanobelts

**DOI:** 10.1007/s40820-019-0300-2

**Published:** 2019-08-26

**Authors:** Wenyu Zhang, Shuquan Liang, Guozhao Fang, Yongqiang Yang, Jiang Zhou

**Affiliations:** 1 School of Materials Science and Engineering, Central South University, Changsha, 410083 Hunan People’s Republic of China; 2 Key Laboratory of Electronic Packaging and Advanced Functional Materials of Hunan Province, Central South University, Changsha, 410083 Hunan People’s Republic of China

**Keywords:** Aluminum vanadate, Graphene, Cathode, High mass loading, Aqueous zinc-ion battery

## Abstract

Rechargeable aqueous zinc-ion batteries (AZIBs) have their unique advantages of cost efficiency, high safety, and environmental friendliness. However, challenges facing the cathode materials include whether they can remain chemically stable in aqueous electrolyte and provide a robust structure for the storage of Zn^2+^. Here, we report on H_11_Al_2_V_6_O_23.2_@graphene (HAVO@G) with exceptionally large layer spacing of (001) plane (13.36 Å). The graphene-wrapped structure can keep the structure stable during discharge/charge process, thereby promoting the inhibition of the dissolution of elements in the aqueous electrolyte. While used as cathode for AZIBs, HAVO@G electrode delivers ideal rate performance (reversible capacity of 305.4, 276.6, 230.0, 201.7, 180.6 mAh g^−1^ at current densities between 1 and 10 A g^−1^). Remarkably, the electrode exhibits excellent and stable cycling stability even at a high loading mass of ~ 15.7 mg cm^−2^, with an ideal reversible capacity of 131.7 mAh g^−1^ after 400 cycles at 2 A g^−1^.

**Electronic supplementary material:**

The online version of this article (10.1007/s40820-019-0300-2) contains supplementary material, which is available to authorized users.

## Highlights

- We first report on H_11_Al_2_V_6_O_23.2_ with large layer spacing as cathode for aqueous zinc-ion battery, which accelerates the diffusion of Zn^2+^.
- The graphene-wrapped H_11_Al_2_V_6_O_23.2_ nanobelts can improve electronic conductivity, and potentially inhibit the dissolution of elements in the aqueous electrolyte.
- H_11_Al_2_V_6_O_23.2_@graphene exhibits high capacity and stable cycling stability even at an ultra-high mass loading of ~ 15.7 mg cm^−2^.

**Electronic supplementary material:**

The online version of this article (10.1007/s40820-019-0300-2) contains supplementary material, which is available to authorized users.

## Graphical abstract

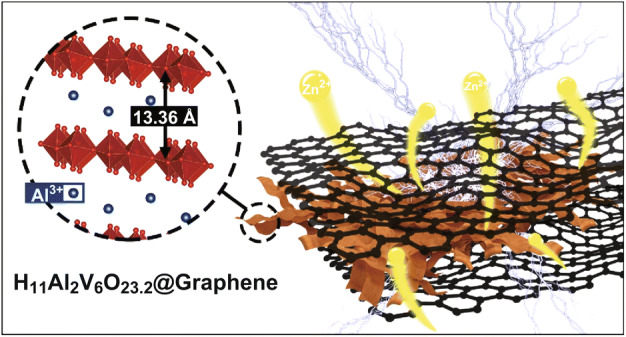

## Introduction

Nowadays, lithium-ion batteries (LIBs) are widely used as energy storage/supply devices in various applications such as portable devices, transportation, and even the military [1–4]. However, the limited reserves of lithium are gradually becoming a non-negligible problem [5]. Their toxic organic electrolytes also cause safety and environmental problems during production and recycling [6]. Hence, new battery chemistries taking into account economic, safety, and environmental issues urgently need to be developed [7–11]. Among them, rechargeable aqueous battery systems using chemically stable, well-stocked multivalent metals (Mg, Zn, Ca, Al, etc.) as anodes and nontoxic aqueous solutions as electrolytes have been widely reported and considered as alternatives to LIBs because of their simple assembly, cost efficiency, high safety, and eco-friendliness [12–15]. The metal Zn, in particular, has a lower standard hydrogen potential [− 0.76 V vs. that of the standard hydrogen electrode (SHE)], and higher theoretical and volume-specific capacities (820 and 5885 mAh cm^−3^, respectively) and chemical stability in aqueous solution, causing aqueous zinc-ion batteries (AZIBs) to attract more attention [16]. However, compared with Li^+^, bivalent Zn^2+^ will enhance electrostatic interaction with the traditional cathodes for LIBs, making them unable to meet the requirements for AZIBs [17]. Finding a cathode that maintains a stable structure during the rapid (de)intercalation of Zn^2+^ becomes the primary problem for AZIBs [18].

In recent years, Prussian blue analogues (PBAs), manganese-based materials, and vanadium-based materials have been successively reported as cathodes for AZIBs and have exhibited their own advantages [19–21]. Manganese-based materials with different crystal structures or valence states of Mn, such as *α*-MnO_2_, *β*-MnO_2_, *γ*-MnO_2_, and Mn_2_O_3_, have been researched and found to reflect higher and smooth operating voltages but poor reversible capacities [22–26]. On the other hand, vanadium oxides (V_2_O_5_, VO_2_) are frequently reported to provide ideal capacities, but the dissolution of vanadium in electrolyte and their structural instability inevitably have a negative effect on the cycling stability [27–29]. V_2_O_5_ with layered structure has the ability of storing metal ions [30, 31]. However, because of its narrow inner structure (the interlayer spacing of (001) plane is about 4.4 Å) and poor electronic conductivity, it is still unsuitable for the storage of Zn^2+^.

Through structural modifications, the electrochemical performance of this kind of material has been greatly improved. V_2_O_5_·*n*H_2_O with water molecule intercalated exhibited ideal reversible capacity because of its spacious interior [32], and together with the intercalation of metal ions (such as Na_0.33_V_2_O_5_, Mg_*x*_V_2_O_5_·*n*H_2_O, Li_*x*_V_2_O_5_·*n*H_2_O, Ca_0.25_V_2_O_5_·*n*H_2_O, and Zn_0.25_V_2_O_5_·*n*H_2_O) [33–39], the inner structural stability and electronic conductivity can be improved. Meanwhile, some vanadates, such as LiV_3_O_8_, NH_4_V_4_O_10_, and Na_1.1_V_3_O_7.9_, have also been applied to AZIBs and exhibited desirable properties [40, 41]. With more AZIB, cathodes with larger lattice spacing of specific crystal planes being reported, researches focusing on the layered design of vanadium-based materials are still underway. Bivalent Zn^2+^ is known to have a strong electrostatic interaction with the cathode host. Therefore, the large diffusion channel will facilitate Zn^2+^ diffusion during charge/discharge process, leading to enhancement of cycling stability and rate capability. However, the largest interlayered spacing of crystal planes in these reported vanadium-based cathode materials are generally below 13.0 Å, and the use of trivalent metal ions (Al^3+^, etc.) to modify the structure of vanadium oxides for AZIBs is rarely discussed. In addition, a cause for concern with respect to practical application is that the electrochemical performance is evaluated under high mass loading (e.g., high areal capacity), which is critical for achieving cell-level energy and power density. Unfortunately, there is very little discussion about this aspect on previously reported AZIBs.

In this work, we have, for the first time, synthesized graphene-wrapped H_11_Al_2_V_6_O_23.2_ nanobelt (HAVO@G) composites through a hydrothermal method and a further freeze-drying treatment. The large spacing of (001) planes and loose arrangement of the nanobelts in HAVO@G provide an ample inner structure and external contact area for the electrochemical reaction, and the presence of Al^3+^ may enhance the electronic conductivity. It is found that the nanobelt morphology could be completely preserved and uniformly coated by the graphene during discharge/charge. Meanwhile, it cannot be ignored that the surface coating of graphene in HAVO@G may inhibit the dissolution of elements in the electrolyte. While used as cathode for AZIBs, HAVO@G electrode exhibits excellent and stable rate performance (delivers average reversible capacities of 305.4, 276.6, 230.0, 201.7, and 180.6 mAh g^−1^ at current densities from 1 to 10 A g^−1^, respectively). Importantly, even at a high loading mass of ~ 15.7 mg cm^−2^, the composite also performs at an ideal reversible capacity and cycling performance (131.7 mAh g^−1^ after 400 cycles at 2 A g^−1^).

## Experimental Section

### Synthesis of HAVO@G

Typically, 2.0 mmol V_2_O_5_, 1.3 mmol AlCl_3_·6H_2_O, 75 mg graphene oxides (prepared using an improved Hummers method [42]), and 2.0 mL 30% H_2_O_2_ solution were dissolved in 25 °C deionized water (30 mL) with vigorous stirring for 4 h. The solution was then transferred into a 50-mL Teflon-lined autoclave and maintained at 180 °C for 12 h. After cooling to indoor temperature (20 °C), the composite was washed repeatedly with deionized water and freeze-dried for 48 h after the pre-freezing.

### Material Characterization

X-ray diffraction (XRD) patterns were measured using Rigaku D/max 2500 X-ray powder diffractometer with Cu Kα-radiation (λ = 0.15405 nm). The morphology was displayed via scanning electron microscopy (SEM, FEI Nova Nano SEM). Transmission electron microscopy (TEM, Tecnai G2 F20) was used to investigate high-resolution TEM (HRTEM) images, selected area electron diffraction (SAED) patterns, and energy dispersive spectrometer (EDS) element mappings. X-ray photoelectron spectroscopy (XPS) spectra were collected through an ESCALAB 250 Xi X-ray photoelectron spectrometer (Thremo Fisher). Differential scanning calorimetry (DSC) and thermogravimetric (TG) analysis curves were collected by the instrument (Netzsch STA449 C, Germany) in air at a heating ramp rate of 10 ^◦^C min^−1^.

### Electrochemical Measurements

Stainless coin cells (CR2016), with metal zinc as anode, glass fiber as separator, and 2 M ZnSO_4_ solution as electrolyte, were assembled in air to investigate the electrochemical performance of the HAVO@G cathode, which was prepared by coating a slurry mixed with the active material (HAVO@G, 70 wt%), acetylene black (20 wt%), and polyvinylidene fluoride (PVDF, 10 wt%) with *N*-methyl-2-pyrrolidone (NMP) onto a stainless steel wire mesh (SSWM), and drying in a vacuum oven at 80 °C for 12 h.

The electrochemical performances of the Zn//HAVO@G cells were all measured in the voltage range of 0.4–1.4 V (vs. Zn^2+^/Zn). Cyclic voltammetry (CV) at different scan rates were carried out using CHI-660E electrochemical station. The galvanostatic intermittent titration technique (GITT) measurement was taken in Arbin Battery Tester BT-2000 (Arbin Instruments, Inc., College Station, Texas). The specific capacities of HAVO@G and H_11_Al_2_V_6_O_23.2_ (HAVO) electrodes were calculated based on the weights of HAVO@G and HAVO, respectively.

## Results and Discussion

**Fig. 1:**
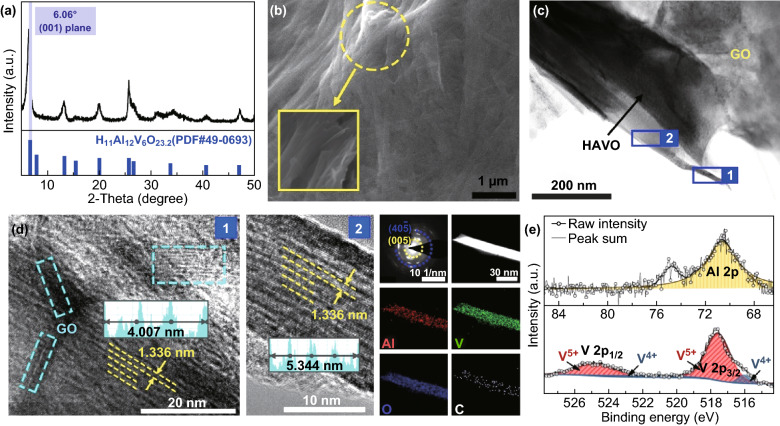
**a** XRD pattern, **b** SEM image, **c** TEM image, **d** HRTEM images with SAED pattern and TEM-EDS element mappings, and **e** High-resolution XPS spectra with Al and V elements of HAVO@G

The XRD pattern of HAVO@G is shown in Fig. 1a. It can be observed that all the diffraction peaks are indexed to the monoclinic crystalline phase of H_11_Al_2_V_6_O_23.2_ [PDF#49-0693] without any impurity. Particularly, a strong peak located at 6.61° corresponds to the (001) crystal plane in the composite, and its lattice spacing calculated according to Bragg’s law is 13.36 Å, which is 3 times larger than the original layered structure of V_2_O_5_ (4.4 Å, PDF#41-1426), and basically larger than other vanadium-based cathodes (such as VO_1.52_(OH)_0.77_ and (NH_4_)_2_V_10_O_25_·8H_2_O) reported previously [43, 44]. Such tremendous interlayer spacing of (001) plane is achieved thanks to the incorporation of Al^3+^ in the layered structure of vanadium oxides [35, 45]. On the other hand, SEM images reflect that the HAVO@G nanobelts, which are generally about 3 μm in length and 0.5 μm in width, are completely covered by graphene with smooth surface (Fig. 1b). The TEM image also indicates that the HAVO nanobelts are coated by the graphene (Fig. 1c). This is further confirmed by the HRTEM image (Fig. 1d), which shows a series of lattice fringes of graphene (green dotted box) clearly observed on the surface of the HAVO nanobelt in region 1. The Raman spectrum of HAVO@G shows two characteristic bands located at around 1367 and 1597 cm^−1^ for D-band and G-band, respectively (Fig. S1), which further indicates the existence of graphene. In addition, the lattice spacing (1.336 nm) corresponds to the (001) plane of the HAVO phase, which is consistent with the results in the XRD pattern. The diffraction rings in the SAED pattern reflect the high-angle (005) and (40$\bar{5}$) planes of HAVO, respectively. In the meantime, the homogeneous distribution of Al, V, and O elements in the nanobelts can be seen in the TEM-EDS element mappings. Furthermore, XPS spectra (Fig. 1e) were used to ascertain the elemental composition of HAVO@G, in which the Al *2p* peak located at 70.7 eV is associated with the oxidation degree of Al^3+^. In addition, the peaks located at 517.6 and 524.9 eV correspond to V *2p*_3/2_ and V *2p*_1/2_ core levels of V^5+^, respectively, while the peaks at 516.0 and 523.1 eV are ascribed to V *2p*_3/2_ and V *2p*_1/2_ core levels of V^4+^, respectively. The result reflects the mixed valence state of vanadium species existing in HAVO@G. Hence, it can be believed that the relatively roomy internal space supported by the (001) planes, together with the enhanced electronic conductivity assisted by the existing Al^3+^, mixture of vanadium valence states of V^5+^ and V^4+^, and the graphene-coated structure, gives HAVO@G potential for the storage of Zn^2+^ [46, 47].

The TG and DSC curves (Fig. S2) are further collected with a temperature ramp rate of 10 °C min^−1^ in air to determine the proportion of graphene oxide (GO) and HAVO in HAVO@G composites. Two main weight loss stages observed on the TG curves result in a weight loss of 23.09%. The first one before 140 °C can be attributed to the decomposition of water from H_11_Al_2_V_6_O_23.2_, corresponding with the endothermic peaks at 78.6 and 111.5 °C in the DSC curve, while the last one is due to the decomposition of graphene and oxidation of vanadium to form AlV_3_O_9_ [48]. We calculated the proportion of GO and HAVO in the HAVO@G composites based on the conserved molar quantity of aluminum, which shows the proportion of GO is about 11.9% in the HAVO@G composites.

**Fig. 2:**
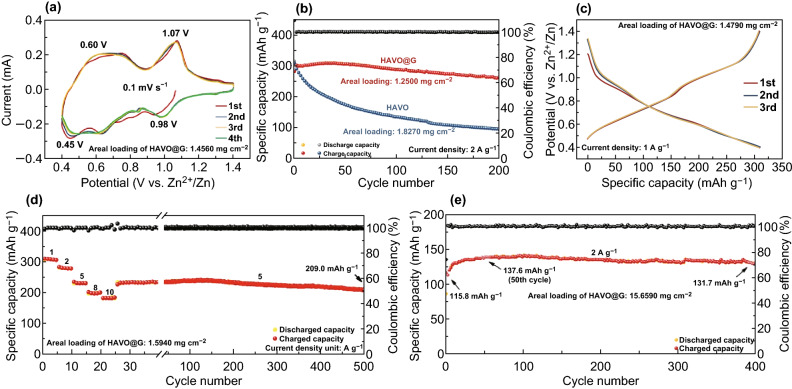
**a** CV curves at a scan rate of 0.1 mV s^−1^ for HAVO@G. **b** Cycling performances at a current density of 2 A g^−1^ of HAVO and HAVO@G. **c** Galvanostatic charge–discharge curve at 1 A g^−1^ for HAVO@G. **d** Rate capability at 1–10 A g^−1^ of HAVO@G. **e** Cycling performance at 2 A g^−1^ of HAVO@G with high areal loading

The electrochemical performances of Zn//HAVO@G AZIBs have been investigated using 2 M ZnSO_4_ aqueous solution as the electrolyte. CV curves at 0.1 mV s^−1^ have been measured to reflect the Zn^2+^ (de)intercalation process in HAVO@G at the beginning cycles (Fig. 2a). Two pairs of redox peaks, located at 1.07/0.98 V and 0.60/0.45 V, respectively, demonstrate a multistep (de)intercalation of Zn^2+^ in HAVO@G, which is frequently reported in other vanadium-based cathodes [32, 35]. Furthermore, the stable position of redox peaks and the substantially unchanged area of the closed portion in the curves demonstrate the high reversibility of the electrode. The galvanostatic charge–discharge (GCD) measurement at a current density of 2 A g^−1^ has been taken to preliminary investigate the electrochemical performance of HAVO@G, as shown in Fig. 2b. HAVO prepared without adding graphene is used for comparison. The HAVO@G exhibits a more ideal reversible capacity (280.2 mAh g^−1^ after 200 cycles), while significant capacity fading can be observed in HAVO electrode, which may be due to the partial dissolution of the active material and poor electronic conductivity [21, 49]. It is reported that NaV_3_O_8_·1.5H_2_O nanobelts exhibited rapid capacity fading due to the fast dissolution of NaV_3_O_8_·1.5H_2_O in the aqueous ZnSO_4_ electrolyte [21]. Dissolution of the active material leading to capacity fading was also observed for manganese-based oxides [50, 51]. For example, Liu et al. demonstrated rapid deterioration in capacity for α-MnO_2_ in 2 M ZnSO_4_ due to the Mn^2+^ dissolution from the MnO_2_ electrode [50]. It is also reported that graphene scroll-coated *α*-MnO_2_ effectively increases the electrical conductivity and relieves the dissolution of *α*-MnO_2_ during cycling [52]. Therefore, it is reasonable that surface coating graphene plays an important role in cyclic stability of HAVO@G. Meanwhile, the addition of graphene increases the electronic conductivity of HAVO@G (Fig. S3), which may promote the reaction kinetics and diffusion of Zn^2+^, thus leading to excellent rate capability of HAVO@G. The GCD curves at 1 A g^−1^ demonstrate the discharge/charge platforms of HAVO@G (Fig. 2c), which are consistent with the CV results. As shown in Fig. 2d, average reversible capacities of 305.4, 276.6, 230.0, 201.7, and 180.6 mAh g^−1^ at 1, 2, 5, 8, and 10 A g^−1^, respectively, can be observed. When the current density returns to 5 A g^−1^, the HAVO@G electrode can still deliver a reversible capacity of 228.5 mAh g^−1^ and remain stable for 500 cycles (209.0 mAh g^−1^ in the 500th cycle).

Importantly, cycling performances of different areal-mass-loading electrodes have been carried out to explore the potential practical application of HAVO@G. The SEM images of the HAVO@G electrodes indicate that the outline of stainless-steel welded mesh (SSWM) can be seen in the electrode with low areal loading, but it is not visible at the high areal loading one (Fig. S4). It is also obvious that the active material is tightly attached to the SSWM, which facilitates the long-term cycling performance. The cycling performances of HAVO@G with different areal mass loadings at 2 A g^−1^ were tested (Fig. S5). As the areal mass loading increases, the specific capacity decreases, which may be due to underutilization of active materials. It can be also seen from Fig. S6 that the electrochemical impedance value gradually increases with the increase in areal mass loading. Fortunately, all these electrochemical impedance values are lower than that of HAVO with a mass loading of 5.1 mg cm^−2^ (Fig. S3). The HAVO@G electrodes with different areal mass loadings exhibit excellent stability. An electrode with a high mass loading of ~ 15.7 mg cm^−2^, especially, delivers an initial capacity of 115.8 mAh g^−1^ at 2 A g^−1^, which gradually increases to 137.6 mAh g^−1^ in the 50th cycle because of the slow activation process and electrolyte penetration. The electrode maintains 131.7 mAh g^−1^ in the 400th cycle with a capacity retention of 95.7%, based on the maximum capacity (Fig. 2e). Furthermore, HAVO@G with a high areal mass loading of 5.4 mg cm^−2^ exhibits excellent cycling stability at a high current density of 5 A g^−1^, which maintains a capacity retention of 94.0% after 900 cycles, based on the maximum capacity (Fig. S7).

**Fig. 3:**
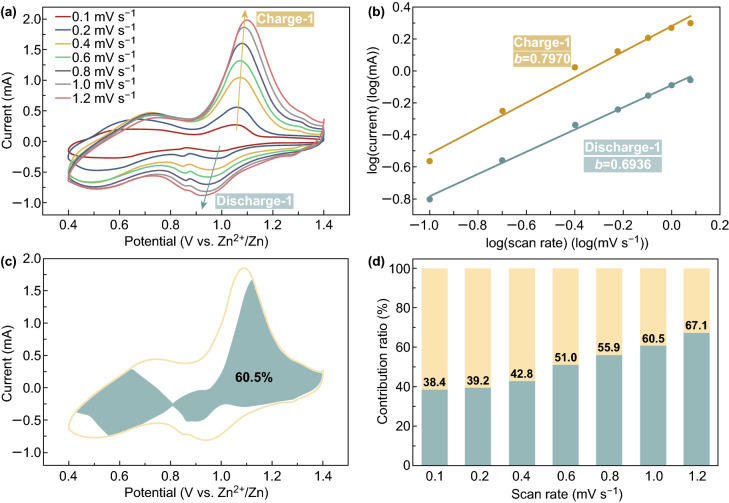
**a** CV curves at 0.1–1.2 mV s^−1^ for HAVO@G. **b** Log(*i*) versus log(*v*) plots at specific peak currents in **a**. **c** CV curve with the calculated pseudocapacitive fraction shown by the shaded area at 1 mV s^−1^, and **d** Bar chart showing the percent of calculated pseudocapacitive contribution at 0.1–1.2 mV s^−1^

To explain the improved electrochemical performance, the CV curves at scan rates between 0.1 and 1.2 mV s^−1^ have been obtained, to investigate the electrochemical kinetics of the HAVO@G, as shown in Fig. 3a. As the scan rate increases, the area of a CV curve with similar shape gradually increases, with the reduction peaks and oxidation peaks shifting to lower and higher voltages, respectively, owing to the polarization effect [53]. In the meantime, the pseudocapacitive characteristic of the HAVO@G electrode can be quantitatively measured by Eqs. 1 and 2 [54]:

1 $$i \, = \, av^{b}$$

2 $$\log \left( i \right) = b\cdot\log \left( v \right) + \log \left( a \right)$$

where *i* is the current (A), *v* is the scan rate (mV s^−1^), and *a* and *b* are adjustable parameters. The value of *b* is from 0.5 to 1, wherein *b *= 0.5 indicates a full diffusion-controlled process and *b* = 1 corresponds to the full capacitive contribution. The *b* values can be obtained by calculating the slope of the log(*i*) *vs*. log(*v*) plots, as shown in Fig. 3b. The *b* values during the discharge and charge processes have been calculated to be 0.69 and 0.80, respectively, demonstrating that the corresponding redox reactions are a combination of the capacitive contribution and ion diffusion process. Thus, the reversible capacity in the cycles can be divided into pseudocapacitive contribution and diffusion contribution according to Eqs. 3 and 4 [55]:

3 $$i = k_{1} v + k_{2} v^{1/2}$$

4 $$i/v^{1/2} = k_{1} v^{1/2} + k_{2}$$

where the pseudocapacitive contribution and the diffusion contribution are measured with *k*_1_ and *k*_2_, respectively. For example, the pseudocapacitive contribution has been calculated to be 60.5% for HAVO@G at 1.0 mV s^−1^, as illustrated by the area with independent color in Fig. 3c. As a whole, a bar chart has been used to show the percent of calculated pseudocapacitive contribution at 0.1–1.2 mV s^−1^, respectively, in which it can be seen that the pseudocapacitive contribution ratios are generally improved from 38.4 to 67.1% with the increase in scan rates (Fig. 3d). The graphene coating and high specific surface area of nanobelt structures cause the pseudocapacitive processes to form majority of the charge storage in HAVO@G, thus exhibiting fast electrochemical reaction kinetics [56].

**Fig. 4:**
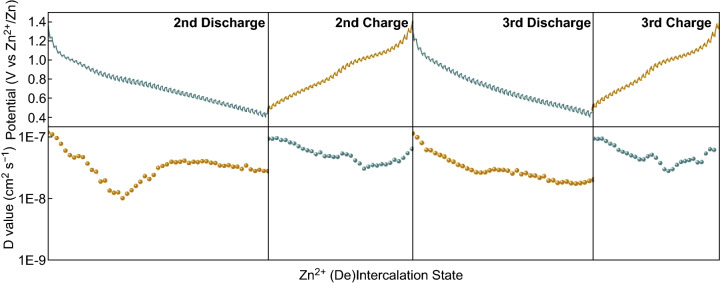
Charge–discharge curves in the galvanostatic intermittence titration techniques (GITT) measurements (above) and corresponding Zn^2+^ diffusion coefficient at different discharge/charge states (below) of HAVO@G

GITT measurement has been taken to further investigate the kinetics of the Zn^2+^ solid-state diffusion of the HAVO@G electrode, in which the diffusion coefficient (*D*) of Zn^2+^ can be calculated from the parameters and voltage changes during the testing, according to Eq. 5 [57]:

5 $$D = \frac{{4L^{2} }}{\pi \tau }\left( {\frac{{\Delta E_{S} }}{{\Delta E_{t} }}} \right)^{2}$$

where *t* and *τ* represent the duration of current pulse (s) and relaxation time (s), respectively. *L* corresponds to the Zn^2+^ diffusion length (equal to the thickness of electrode, ≈ 0.75 mm). *∆E*_*S*_ and *∆E*_*t*_ are the steady-state voltage change (V) by the current pulse and voltage change (V) during the constant current pulse (eliminating the voltage changes after relaxation time), respectively. The measured GCV curves and calculated results are shown in Fig. 4, in which the diffusion coefficient (*D*) calculated is basically consistent in changing trends, and the value falls between 10^−7^ and 10^−8^ cm^2^ s^−1^, which is superior to those of most of the reported vanadium-based cathodes [8, 49, 56]. The reason for this is that the introduction of Al^3+^ in V–O layers enlarging the (001) plane with spacious inner spacing accelerates the Zn^2+^ diffusion process.

**Fig. 5:**
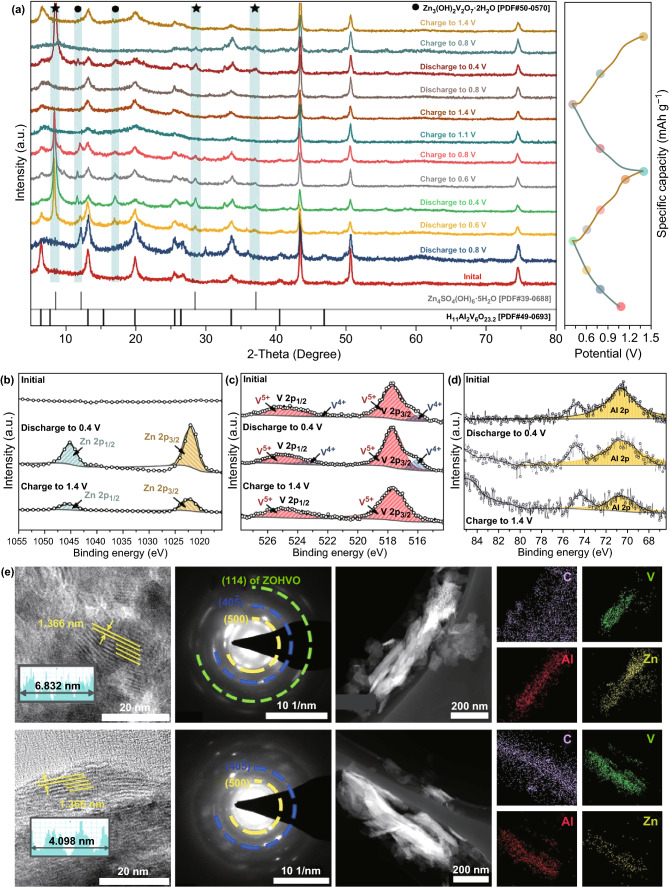
**a** Ex situ XRD patterns and corresponding GCD curves at 0.1 A g^−1^, **b–d** High-resolution XPS spectra of Zn *2p*, V *2p*, and Al *2p* in pristine, fully discharged, and charged states, **e** TEM images, HRTEM images with SEAD patterns, and TEM–EDX element mapping images in fully discharged (above) and charged (below) states of HAVO@G. ZOHVO represents Zn_3_(OH)_2_V_2_O_7_·2H_2_O

The reaction mechanism of the HAVO@G electrode has been explored, to investigate the exceptional performance of the electrode. The ex situ XRD patterns at selected discharged/charged states have been obtained, which reveal that the position of the (001) plane remains unchanged during the cycles but weakens in intensity (Fig. 5a). Meanwhile, some characteristic peaks are observed corresponding to Zn_3_(OH)_2_V_2_O_7_·2H_2_O [PDF#50-0570] in the discharging process, which are due to the intercalated Zn^2+^ bonded with vanadium–oxygen layer to form the new phase. Similar to other reported cathodes for AZIBs, the HAVO@G electrode also exhibits H^+^ intercalation in the discharge process, causing the electrolyte to be alkaline and promoting the formation of Zn_4_SO_4_(OH)_6_·5H_2_O [PDF#39-0688] [58], as indicated in Fig. 5a. Obviously, these two phases disappear upon the subsequent charge process, indicating the highly reversibility of the HAVO@G electrode during the cycles. XPS measurement at full discharged/charged states has been used to analyze the changes in the valence state of elements during the cycles, as shown in Fig. 5b. Two new peaks appear conspicuously at 1022.03 and 1045.02 eV assigned to Zn *2p*_3/2_ and Zn *2p*_1/2_, respectively, when the electrode discharged to 0.4 V, reflecting the existence of Zn^2+^ in HAVO@G. Meanwhile, the signal of V^4+^ intensifies as a consequence of the Zn^2+^ intercalation (Fig. 5c). While charged to 1.4 V, the component of V^4+^ disappears almost with a part of Zn^2+^ remaining in the electrode, which indicates that the V^4+^ that existed in the original substance participates in the electrochemical reaction, leading to an increase in the capacity at the beginning of the cycle. On the other hand, the basically unchanged position and intensity of the characteristic peak located at 70.7 eV determine the stable existence of Al^3+^ in HAVO@G during discharge/charge states (Fig. 5d). Additionally, the TEM image, and the HRTEM image with SEAD pattern have been investigated to intuitively evaluate the evolution of the cycle (Fig. 5e). The (001) planes are present throughout the process with a stable lattice spacing of 1.336 nm but reduce in sharpness because of the decreased crystallinity caused by the intercalation of Zn^2+^ and formation of intermediate products, which is consistent with the weakening in intensity of the diffraction peak in the ex situ XRD patterns. Furthermore, the high-angle (500) and (40$\bar{5}$) planes remain stable during the cycle, according to their constant diffraction rings in SAED patterns. An extra diffraction ring appears corresponding to the (114) plane of the Zn_3_(OH)_2_V_2_O_7_·2H_2_O phase in the full discharged state. The homogeneous distribution of C, V, and Al in HAVO@G, and the intercalation of Zn^2+^ in the discharged state, and the residual of Zn^2+^ during the charging state are further verified via TEM-EDS element mapping. The morphology and graphene-wrapped structure, especially, remain stable during discharge/charge, indicating the excellent stability in the construction of this unique structure. Based on the aforementioned analysis, the highly expanded (001) plane, with the especially stable inner structure of HAVO@G, is suitable for the insertion/extraction of Zn^2+^ accompanied with H^+^. The graphene-wrapped HAVO nanobelt construction is beneficial to keeping the structure stable during discharge/charge process, thereby inhibiting the dissolution of cathode in the electrolyte. Such a battery system also undeniably exhibits high stability and safety during cycling.

## Conclusions

In summary, H_11_Al_2_V_6_O_23.2_@graphene (HAVO@G) composites have been successfully prepared through a hydrothermal method and a further freeze-drying treatment. The as-prepared HAVO@G with intercalation of Al^3+^ possesses a large lattice spacing (~ 13.36 Å), which may provide a broad channel and spacing for the intercalation of Zn^2+^. Meanwhile, the uniform coating of graphene on the surface of the HAVO nanobelts may enhance electrical conductivity and inhibit the dissolution of the active material in electrolyte. While used as a cathode for AZIBs, HAVO@G delivers stable cycling performance and excellent rate capability. Remarkably, HAVO@G exhibits an ideal performance in the high-areal-loading measurement (~ 15.7 mg cm^−2^), demonstrating its potential practical application in large-scale energy storage.

## Electronic Supplementary Material

Below is the link to the electronic supplementary material.

Supplementary material 1 (PDF 870 kb)
