# Supplementary material for: Ultra-High Mass-Loading Cathode for Aqueous Zinc-Ion Battery Based on Graphene-Wrapped Aluminum Vanadate Nanobelts
Source: Nanomicro Lett. 2019 Aug 26;11:69. doi: 10.1007/s40820-019-0300-2 (PMC7770939; doi:10.1007/s40820-019-0300-2)
Supplement: Supplementary file 1 — Supplementary material 1 (PDF 870 kb) [file 40820_2019_300_MOESM1_ESM.pdf]

Supporting Information for

Ultra-high Mass-loading Cathode for Aqueous Zinc-ion Battery Based on Graphene-wrapped Aluminum Vanadate Nanobelts

Wenyu Zhang[1], Shuquan Liang[1, 2, *], Guozhao Fang[1], Yongqiang Yang[1], Jiang Zhou[1, 2, *]

[1]School of Materials Science and Engineering, Central South University, Changsha 410083, Hunan, People's Republic of China

[2]Key Laboratory of Electronic Packaging and Advanced Functional Materials of Hunan Province, Central South University, Changsha 410083, Hunan, People's Republic of China

*Corresponding authors. E-mail: zhou_jiang@csu.edu.cn (Jiang Zhou); lsq@csu.edu.cn (Shuquan Liang)

Supplementary Figures

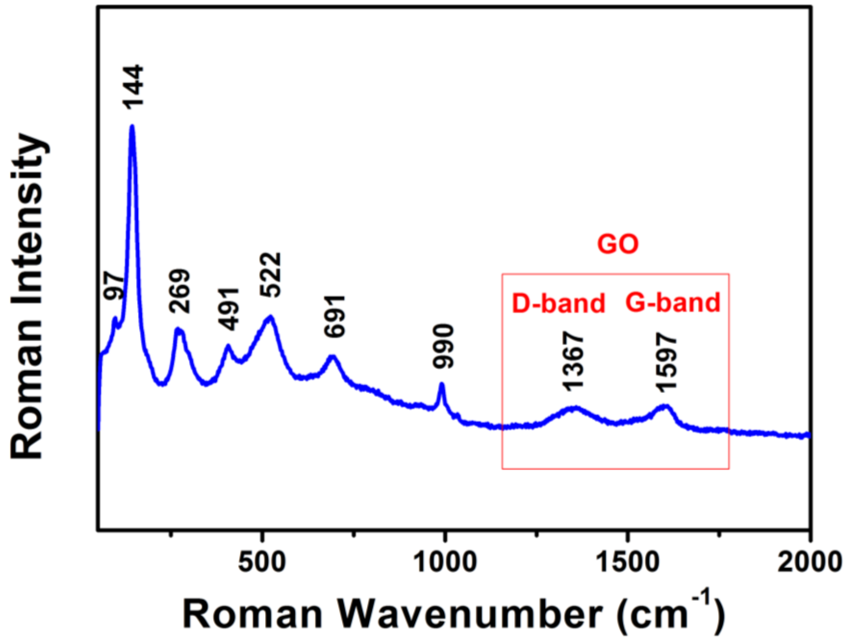

Fig. S1 Raman spectrum of HAVO@G

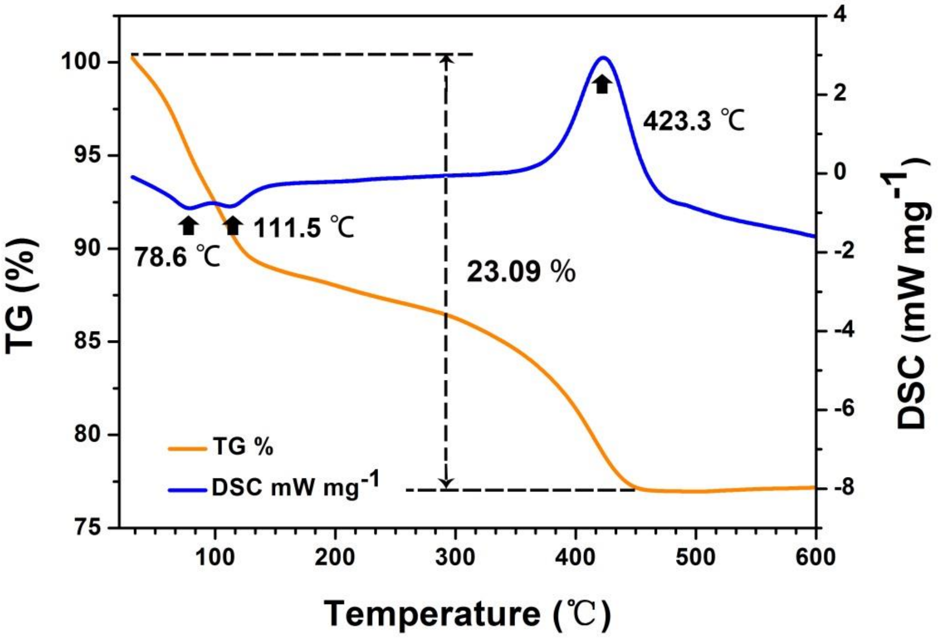

Fig. S2 TG and DSC results of HAVO@G

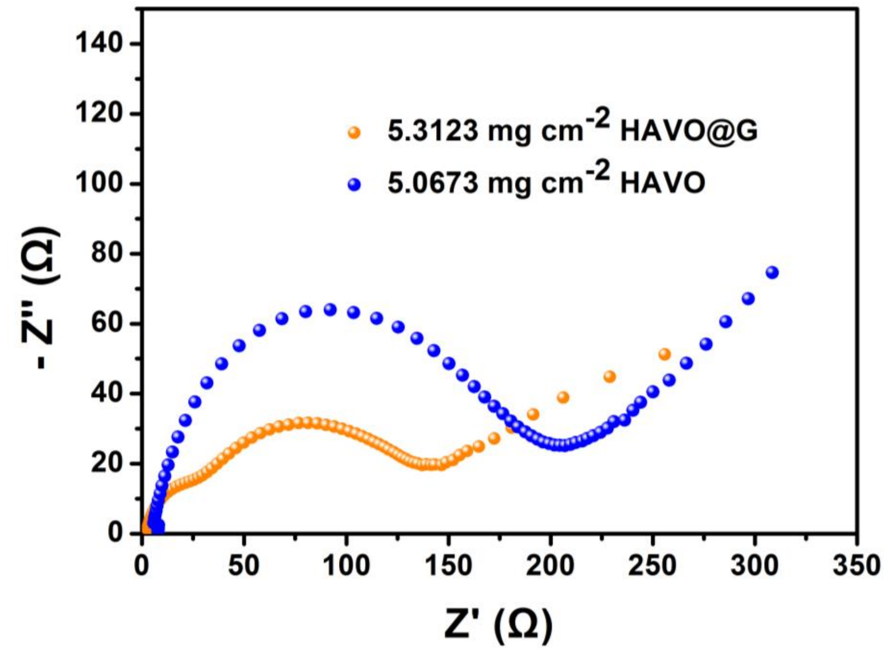

Fig. S3 Electrochemical impedance spectra of HAVO@G and HAVO

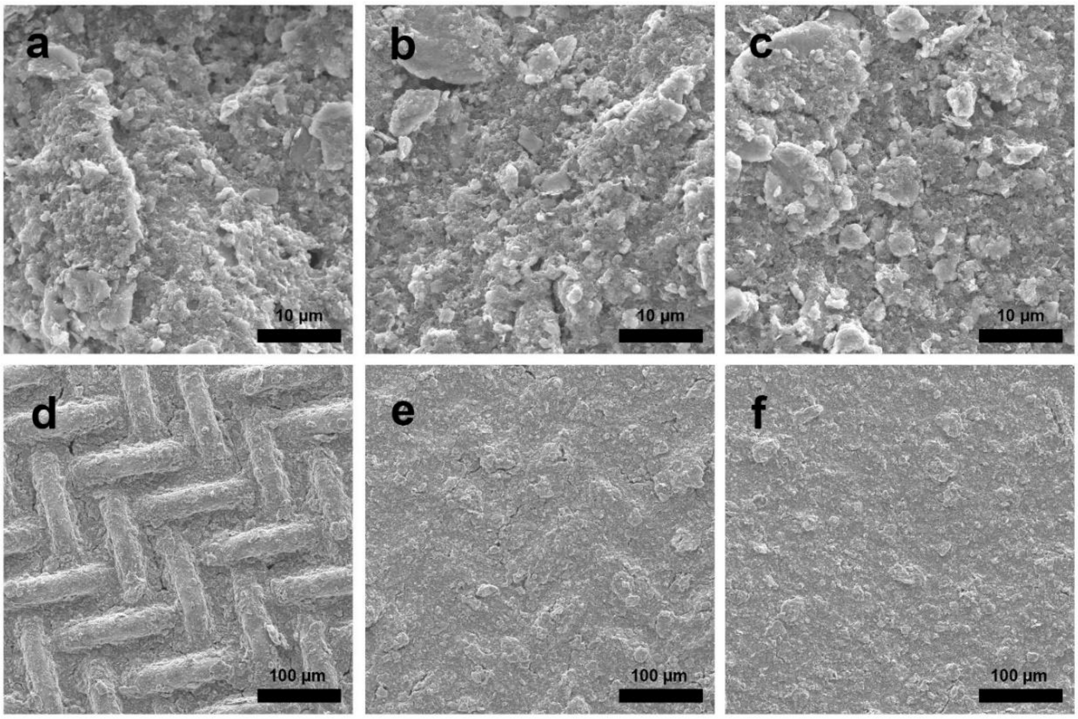

Fig. S4 SEM images of HAVO@G electrodes with different areal loading. (**a, d**) ~ 2 mg cm^{-2}, (**b, e**) ~ 6 mg cm^{-2}, (**c, f**) ~ 12 mg cm^{-2}

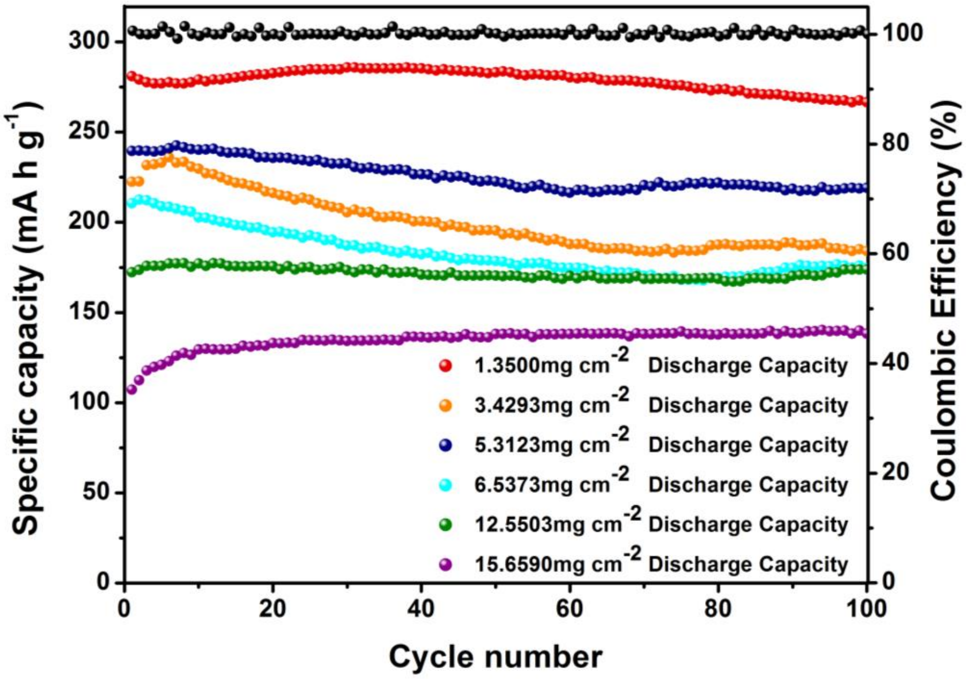

Fig. S5 Cycling performance of HAVO@G with different areal loading at 2 A g^{-1}

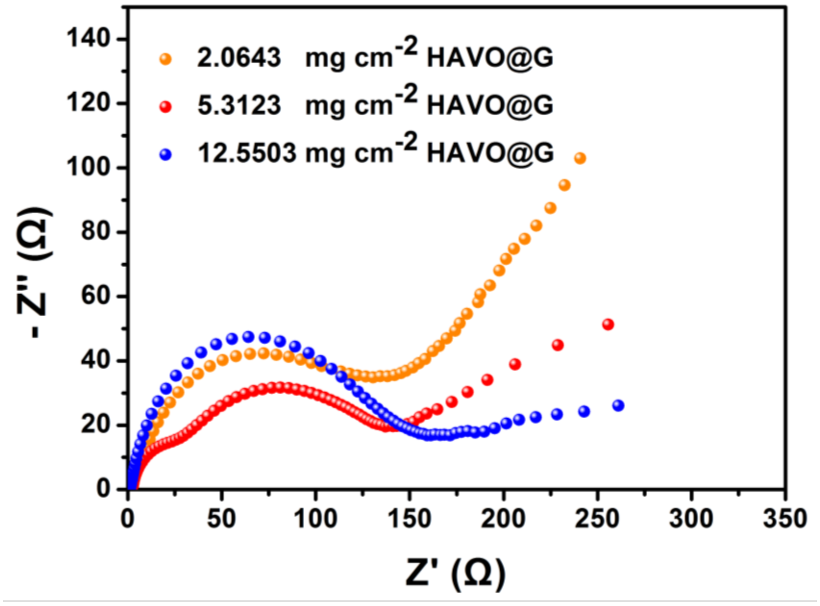

Fig. S6 Electrochemical impedance spectra for HAVO@G with different areal loading

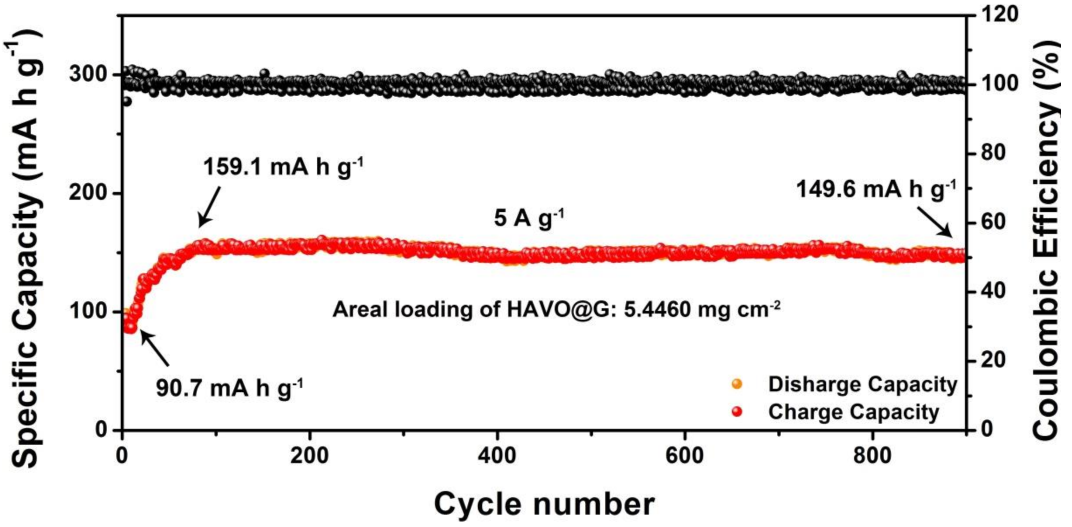

Fig. S7 Cycling performance of HAVO@G at 5 A g^{-1} with a high areal mass loading of 5.4 mg cm^{-2}
